# Risikofaktorenanalyse zu den Gründen des Ausscheidens aus der fachärztlichen Weiterbildung im Fach Orthopädie und Unfallchirurgie

**DOI:** 10.1007/s00113-022-01249-x

**Published:** 2022-11-10

**Authors:** Oskar Brandt, Thorsten Tjardes, Gina Grimaldi, Manuel Mutschler, Sebastian Imach

**Affiliations:** 1 Klinik für Orthopädie, Unfallchirurgie und Sporttraumatologie, Kliniken der Stadt Köln gGmbH, Krankenhaus Köln-Merheim, Universität Witten/Herdecke, Ostmerheimer Str. 220, 51109 Köln, Deutschland; 2 Universitätsklinik für Unfallchirurgie, Otto von Guericke Universität Magdeburg, Magdeburg, Deutschland; 3 Abteilung für Fuß und Sprunggelenkschirurgie, Waldkrankenhaus Bonn, Johanniter GmbH, Universität Witten/Herdecke, Bonn, Deutschland

**Keywords:** Chirurgische Weiterbildungskonzepte, Online-Umfrage, Arbeitszeitrichtlinien, Generation Y, Gleichstellung, Education, Entrustable professional activities (EPA), Survey and Questionnaires, Work-Life Balance, Gender Equity

## Abstract

**Hintergrund:**

Aktuell liegen keine Daten zu Weiterbildungsabbrüchen und Klinikwechseln im Fach Orthopädie und Unfallchirurgie (O&U) vor. Ziele der Studie sind die Identifikation von persönlichen und strukturellen Risikofaktoren, die zum Abbruch/Wechsel der Weiterbildung in O&U führen, sowie Lösungsstrategien vorzustellen.

**Methodik:**

Im Sommer 2020 wurde eine deutschlandweite, anonyme Onlinebefragung unter den Weiterbildungsassistenten*innen (WA) in O&U durchgeführt. Dienstliche Mailadressen wurden über das Traumanetzwerk© der DGU und die Deutsche Krankenhausgesellschaft identifiziert (*n* = 2090). Ein Fragebogen (51 Fragen) wurde mit SurveyMonkey Inc. (San Mateo, California, USA) erstellt. Teilnahmeberechtigt waren alle WA, die in den 6 Jahren vor Umfragebeginn (ab Juli 2014) für mind. einen Monat im Fach O&U tätig waren.

Zur Identifikation der Risikofaktoren wurde eine binär logistische Regression berechnet. Das Signifikanzniveau lag bei *p* = 0,05.

**Ergebnisse:**

Von den 221 Befragten wechselten 37 % die Weiterbildungseinrichtung, und 5 % brachen die Weiterbildung in O&U vorzeitig ab.

Die Regression ergab 3 signifikante Risikofaktoren, die Klinik zu wechseln.

Das Leben in einer Partnerschaft (*p* = 0,029, RR: 2,823) und weniger als 2 Tage Hospitationen vor Weiterbildungsbeginn (*p* = 0,002, RR: 2,4) erhöhen das Risiko für Wechsel. Eine Einteilung der WA für Operationen gemäß dem Weiterbildungsplan/-stand (*p* = 0,028, RR: 0,48) senkt das Risiko für Wechsel. Signifikante Risikofaktoren für das Ausscheiden aus der Weiterbildung konnten nicht ermittelt werden (zu geringe Fallzahl, *n* = 11).

**Diskussion:**

Weiterbildungswechsel und -abbrüche in der O&U sind ein relevantes Problem (42 %). Das Geschlecht hat keinen signifikanten Einfluss. Maßnahmen wie längere Hospitationen sowie Op.-Einteilung entsprechend dem Weiterbildungsplan/-stand können das Wechselrisiko minimieren.

**Graphic abstract:**

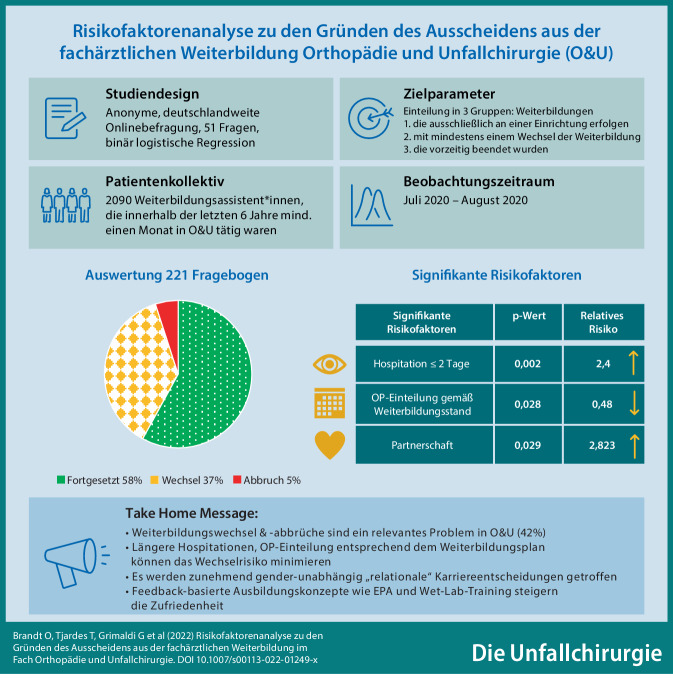

**Zusatzmaterial online:**

In der Online-Version dieses Beitrags (10.1007/s00113-022-01249-x) finden Sie den in der Studie verwendeten Fragebogen.

## Abstract (en)

**Background:**

Currently, there are no data available on dropouts from residency programs and changes of clinic in orthopedics and trauma surgery (O&T). The aim of the study is to identify personal and structural risk factors leading to dropout or switching of postgraduate training in O&T in order to present solution strategies.

**Methods:**

A nationwide anonymous online survey was conducted among residents in O&T in summer 2020. Official mail addresses were identified via the Traumanetzwerk© of the DGU and the German Hospital Federation (*n* = 2090). A questionnaire (51 questions) was administered using SurveyMonkey (San Mateo, CA, USA). All residents who worked in O&T for at least 1 month in the 6 years prior to the start of the survey (from 07/2014) were eligible to participate.

A binary logistic regression was calculated to identify the risk factors. The significance level was *p* = 0.05.

**Results:**

Of the 221 respondents, 37% switched hospital and 5% dropped out altogether.

The regression revealed 3 significant risk factors for switching hospitals.

Living in a partnership (*p* = 0.029, RR: 2.823) and less than 2 days of shadowing before the start of residency (*p* = 0.002, RR: 2.4) increased the risk of switching. Operating room (OR) allocation of residents according to the training plan/status (*p* = 0.028, RR: 0.48) reduces the risk of switching. Significant risk factors for leaving postgraduate training could not be determined (insufficient number of cases, *n* = 11).

**Discussion:**

Switching the hospital and residency dropouts in O&T are a relevant problem (42%). Gender has no significant influence. Tools such as longer job shadowing, as well as OR allocation according to the training plan/status can minimize the risk of switching.

Die **Generation Y **(Jgg. 1981–2000) legt Wert auf eine ausgewogene Balance zwischen Privatleben und Beruf. Mit **zwingend einzuhaltenden Arbeitszeitrichtlinien** führt dies zu vermehrten Weiterbildungswechseln und -abbrüchen. Deshalb muss eine zukunftssichere Weiterbildung in O&U diese neuen Konstanten erfolgreich integrieren.

Während andere chirurgische Fachgebiete dieses Problem erfasst haben, existieren für die O&U bisher keine Daten. Dieser Beitrag stellt individuelle und strukturelle Risikofaktoren für Abbrüche und Wechsel der Weiterbildung in O&U vor und diskutiert mögliche Lösungsstrategien.

## Hintergrund und Fragestellung

Die Rahmenbedingungen der Medizin haben sich in den letzten 20 Jahren grundlegend verändert. Der medizinische Anspruch steht in Konkurrenz zu wirtschaftlichen Notwendigkeiten. Parallele epidemiologische, soziologische und legislative Veränderungen machen auch ein Überdenken der Facharztweiterbildung in O&U erforderlich, um den Versorgungsbedarf des Faches in Zukunft durch einen konstant hohen Prozentsatz von erfolgreich abgeschlossenen Weiterbildungen decken zu können.

1. *Generation Y – „Leben beim Arbeiten“*
  Das** Wertegerüst der Generation Y** beinhaltet den Anspruch, einen Ausgleich zwischen Privat- und Berufsleben zu schaffen, sowie eine geringere Akzeptanz von Hierarchien. Diesen Bedürfnissen wird die Loyalität gegenüber dem*r Arbeitgeber*in untergeordnet, sodass die Hemmschwelle, den Arbeitsplatz zu wechseln, deutlich sinkt [1–3].
2. *Arbeitszeitrichtlinien*
  Die europäische Richtlinie zur Arbeitszeitgestaltung von 2003 sieht eine wöchentliche Höchstarbeitszeit von 48 h vor [4]. Eine regelhafte Erfassung der Arbeitszeiten ist seit Mai 2019 europaweit Pflicht (Gerichtsurteil EuGH) [5]. Es wurde versäumt, die Auswirkungen dieser Regelungen auf die chirurgische Weiterbildung zu berücksichtigen, sodass gegenwärtig die fristgerechte Erfüllung der Weiterbildungskataloge in einem Fach mit hohem Notfallaufkommen, wie der Unfallchirurgie, de facto nicht möglich ist, was zu zusätzlicher Unzufriedenheit bei Weiterbildungsassistent*innen (WA) führt [6].
3. *Prozentualer Zuwachs von Ärztinnen in der chirurgischen Weiterbildung*
  Zwei Drittel der Studienanfänger*innen in der Humanmedizin sind weiblich. Nach Abschluss des Studiums entscheiden sich allerdings nur 17 % der Absolventinnen (vgl. männlich 25 %) für die operativen Fächer [7]. Die berufliche Wunschvorstellung von Studentinnen lässt sich als „Möglichkeit der Familiengründung ohne Verzicht auf Karriere“ zusammenfassen [8]. Weibliche Rollenvorbilder gilt es, als Mentoren zu nutzen und regelmäßige Zwischenvalidierung in der Weiterbildung durchzuführen [9].
  In einer geschlechtsspezifischen Analyse zu den Herausforderungen des unfallchirurgischen Nachwuchses ist das frauenspezifische Alleinstellungsmerkmal die Fähigkeit zu Schwangerschaft und Geburt eines Kindes [10]. Hierbei gilt es, undifferenzierte Berufsverbote während der Schwangerschaft zu vermeiden und in der Folge die „gender care gap“, d. h. die Übernahme von Sorge‑/Care-Arbeit im privaten Umfeld, die in Konkurrenz zur beruflichen Karriere steht, zu minimieren [11]. Eine Individualisierung von Arbeitszeitmodellen mit der Möglichkeit zum Jobsharing, arbeitsplatznahe Kinderbetreuung und gleichberechtigte Aufstiegschancen sind dazu zu fordern.

Die Kündigung eines*einer WA bedeutet für die Klinik einen Produktivitätsverlust vom Zeitpunkt der Kündigung bis zur abgeschlossenen Einarbeitung des*der neuen Mitarbeiters*in. Die Kündigungsfrist und somit der Produktivitätsverlust wachsen mit der Beschäftigungsdauer des*der WA (erstes WB Jahr 6 Wochen, ab dem 5. Jahr 3 Monate) [12, 13]. Diese Überlegungen sind insbesondere vor dem Hintergrund des steigenden Bedarfs an orthopädisch/unfallchirurgischen Leistungen als Folge der demografischen Entwicklung, bei gleichzeitiger Verschärfung des Nachwuchsmangels, von großer Relevanz [7, 14–18]. Die Minimierung von Weiterbildungsabbrüchen/-wechseln ist deshalb ein wesentlicher Beitrag zur Sicherstellung der Versorgung im Fach O&U.

Ziel der vorliegenden Studie ist es, persönliche und strukturelle Risikofaktoren zu identifizieren, welche zum Abbruch bzw. zum Wechsel der Weiterbildung in O&U führen.

## Methodik

Im Sommer 2020 wurde dann eine Online-Umfrage (SurveyMonkey Inc., San Mateo, California, USA) unter den WA im Fach O&U durchgeführt. Der Fragebogen wurde zunächst mit den WA der Klinik des Letztautors validiert (Pretest), da für dieses Kollektiv sämtliche Messparameter zur vergleichenden Auswertung verfügbar waren. Es zeigte sich eine hohe Reliabilität (< 10 % Abweichung). Zur Steigerung der Validität wurde die Formulierung der Fragen 32 und 33 angepasst. Der Survey war danach von Juli 2020 bis August 2020 abrufbar. Ein Reminder wurde 2 Wochen nach erstmaligem Versand und vor Schließung der Umfrage versandt. Es gab neben der Möglichkeit, den Survey nicht zu beantworten, die Option, nur einige Fragen unbeantwortet zu lassen oder die Befragung im Ganzen abzubrechen.

Der Survey umfasst 46 Fragen, die in Abhängigkeit des individuellen Weiterbildungsverlaufs mit 5 zusätzlichen Fragen ergänzt wurden. Es wurden dichotome Fragestellungen (ja/nein), Katalogfragen (Mehrfachantworten) und Likert-Skalen (6 stufig, 1–2 keine Zustimmung, 3–4 schwache Zustimmung, 5–6 starke Zustimmung) verwendet [19].

### Studienziel

Ziel der Datenerhebung war es, eine Datengrundlage zum Verständnis des Wechsel- und Abbruchverhaltens von WA zu schaffen, auf deren Grundlage Maßnahmen abgeleitet werden können, die die Attraktivität einer Weiterbildung in O&U nachhaltig erhöhen. Die Studienkohorte wurde in 3 Gruppen unterteilt:

1. Weiterbildungen, die **ausschließlich an einer** Weiterbildungseinrichtung erfolgen,
2. Weiterbildungen, bei denen **mindestens ein** Wechsel der Weiterbildungseinrichtung erfolgte,
3. Weiterbildungen, die **vorzeitig beendet** wurden.

Im Weiteren wurden diese Gruppen vor dem Hintergrund der im Rahmen einer Literaturrecherche als für einen erfolgreichen Abschluss der Weiterbildung relevant identifizierten Rahmenbedingungen analysiert:

1. persönlich-biografische Umstände [20],
2. persönliche Vorkenntnisse/klare/unklare Erwartungshaltung [21],
3. Arbeitsbedingungen in der Klinik [3],
4. Weiterbildungsstruktur [22].

### Studienkohorte

Im öffentlich zugänglichen Klinikverzeichnis der Traumanetzwerke© der DGU und einer digitalen Mitgliedsliste der Deutschen Krankenhausgesellschaft wurden 2090 WA identifiziert. Zur Teilnahme zugelassen wurden WA, die ab Juli 2014 für mindestens einen Monat in der O&U tätig waren.

Um den Einfluss der einzelnen Variablen auf Abbruch oder Wechsel der Weiterbildung zu ermitteln, wurden die jeweiligen Risiken (absolutes und relatives Risiko, Konfidenzintervall (KI) 95 %) mit binär logistischen Regressionsmodellen berechnet. Für die in der Regressionsanalyse signifikanten Risikofaktoren wurde das relative Risiko durch eine univariate Analyse ermittelt.

Für kategorische Variablen wurden die totale Anzahl und die Prozentwerte angegeben, für kontinuierliche Variablen wurden Durchschnittswerte und die Standardabweichung angegeben. Das Niveau für den α‑Fehler wurde auf 5 % (*p* *<* *0,05*) festgelegt.

Analysiert wurden sowohl vollständig ausgefüllte als auch teilweise ausgefüllte Fragebogen, wobei Fragebogen mit < 15 % beantworteten Fragen von der Auswertung ausgeschlossen wurden.

Für die Datenanalyse wurden die Tabellenkalkulationsprogramme MS Excel 365° (Microsoft Inc., Redmond, WA, USA) und SPSS Version 27.0 (International Business Machines Corporation (IBM Corp.), Armonk, NY, USA) genutzt.

## Ergebnisse

**Figure 1:**
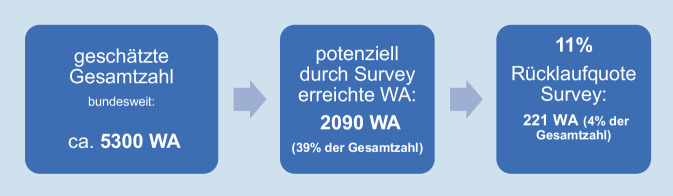

Über zweihundertfünfzig (253) von 2090 WA beantworteten den Survey (Bruttorücklaufquote 12 %), davon waren 32 Fragebogen unvollständig (< 15 % beantwortete Fragen). Es wurden daher 221 Datensätze ausgewertet (Nettorücklaufquote 11 %; Abb. 1).

### Demografie

Die WA waren im Median 28 Jahre alt (Min/Max 24/41) bei einem mit 48 %/52 % (w/m) ausgeglichenen Geschlechterverhältnis. 54 % der Teilnehmer*innen befanden sich im 3. bis 6. Ausbildungsjahr („special trunk“), 30 % im 1. bis 2. Ausbildungsjahr („common trunk“), und 16 % waren bereits Fachärzt*innen. Fachärzt*innen wurden explizit nur zu ihrem Wechselverhalten während der Facharztweiterbildung befragt (Zusatzmaterial online: Frage 31 des Survey). Die durchschnittliche Anzahl an WA an den Kliniken der Befragten WA betrug 20.

### Weiterbildungsverhalten

Die Fragen zum Weiterbildungsverhalten wurde von allen WA (*n* = 221) beantwortet. 128 WA (58 %) haben die Weiterbildungseinrichtung nicht gewechselt, 82 WA (37 %) haben die Klinik mindestens einmal gewechselt, und 11 WA (5 %) haben die Weiterbildung vorzeitig beendet.

143 WA (65 %) gaben, an Wechsler*innen an ihrer Weiterbildungsstätte persönlich zu kennen, und 122 WA (55 %) gaben an, Abbrecher*innen an ihrer Weiterbildungsstätte persönlich zu kennen. Die Befragten gaben an, in ihrem beruflichen Umfeld durchschnittlich von 4 Wechseln der Weiterbildungseinrichtung und 3 Weiterbildungsabbrüchen Kenntnis erlangt zu haben.

### Bewertung der Rahmenbedingungen

**Figure 2:**
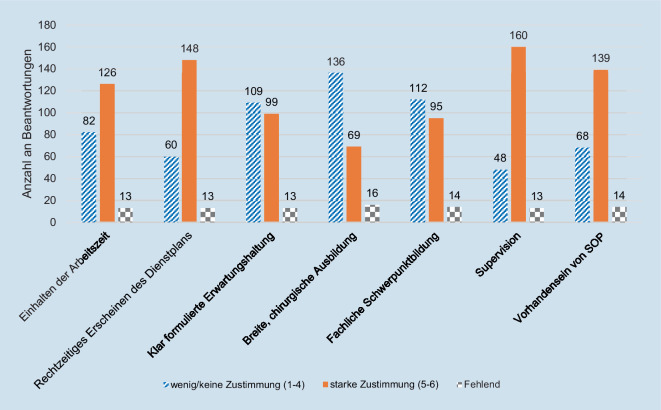

Der Median der Geburtsjahrgänge der Studienteilnehmer*innen ist das Jahr 1992, was sie zentral in die Geburtsjahrgänge der Generation Y verortet. Aus der Auswertung der Likert-Skalen ergab sich, dass mehr als die Hälfte der Befragten das Einhalten der Arbeitszeiten, das rechtzeitige Erscheinen des Dienstplanes, die direkte, persönliche Anleitung bei Tätigkeiten (Supervision) und das Vorhandensein klinikinterner Standards (SOP) zur Versorgung von Patient*innen für sehr wichtig erachten (starke Zustimmung) (Abb. 2). In der Gruppe der Wechsler*innen erachten signifikant mehr WA das Einhalten von Arbeitszeiten als wichtig (62 %; 51/82; *p* = 0,03).

**Figure 3:**
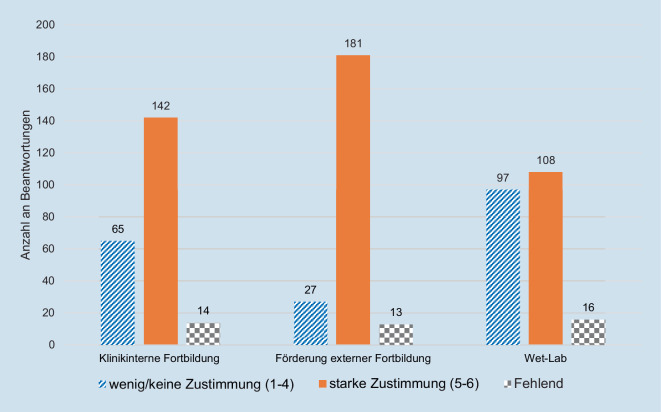

Klinikinterne genauso wie klinikexterne Fortbildungsmöglichkeiten erachteten mehr als 60 % für wichtig. Die zeitliche und/oder finanzielle Förderung von klinikexternen Fortbildungsveranstaltungen empfanden 82 % (181/221) als wichtig (Abb. 3).

**Figure 4:**
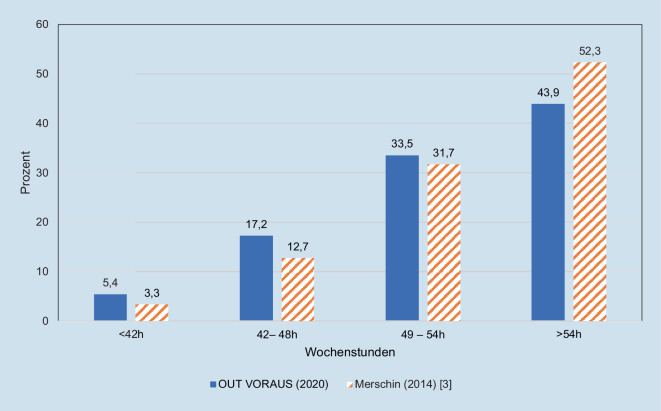

Wöchentlich arbeiten 23 % durchschnittlich 48 h oder weniger (EU-Arbeitszeithöchstgrenze [4]). Im Vergleich zu den aus dem Jahr 2014 in Deutschland erhobenen Daten zeigt sich keine relevante Veränderung (Abb. 4; [3]).

### Gründe für den Abbruch oder den Wechsel der Weiterbildung

**Figure 5:**
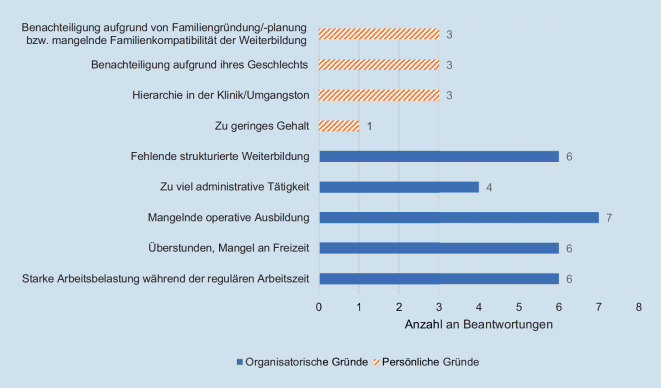

**Figure 6:**
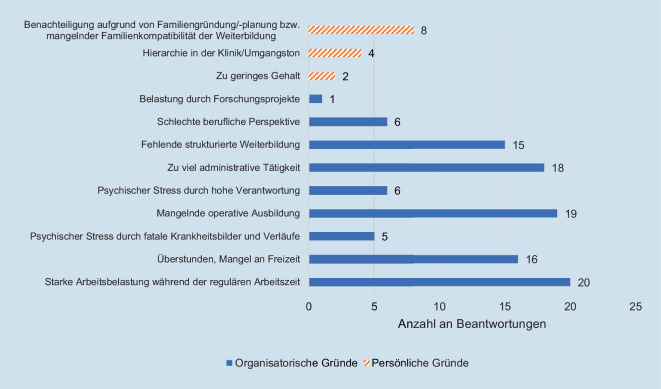

45 % (5/11) der Weiterbildungsabbrüche fanden im 3. Ausbildungsjahr (Übergang vom Common trunk zum Special trunk) statt. 90 % der Kündigungen erfolgten fristgerecht und geplant, mit einer konkreten, weiteren beruflichen Perspektive. Ausbildungsabbrüche werden gleichermaßen mit persönlichen wie mit strukturellen Mängeln begründet (Abb. 5), wohingegen die Motivation zum Wechsel der Weiterbildungseinrichtung mehrheitlich mit strukturellen Mängeln begründet wird (Abb. 6).

### Binär logistische Regression der Risikofaktoren für einen Klinikwechsel

**Figure 7:**
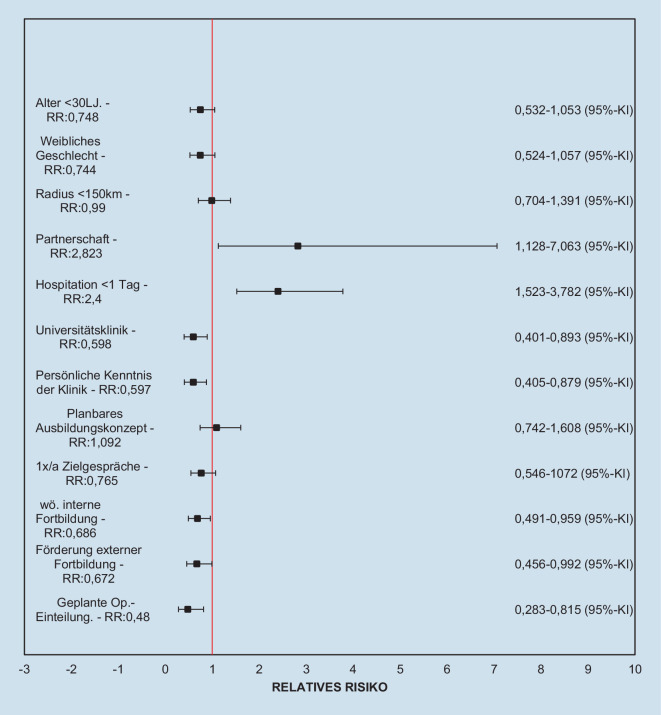

Ein Regressionsmodell wurde für die potenziellen Risikofaktoren Alter, Geschlecht, Nähe des Arbeitsplatzes zum Heimatort, Partnerschaft, Hospitation vor Weiterbildungsbeginn, Einrichtung, in der die Weiterbildung begonnen wurde, Grund für die Auswahl der bestimmten Einrichtung, strukturiertes Ausbildungsprogramm, stattfindende Zielvereinbarungsgespräche, klinikinterne Fortbildungsveranstaltungen, Förderung klinikexterner Fortbildungsveranstaltungen und Faktoren, die zur Op.-Einteilung führen, berechnet (Abb. 7).

Das binär logistische Regressionsmodell war statistisch signifikant, χ^2^ (12) = 50,753, *p* < 0,001, mit einer akzeptablen Varianzaufklärung von Nagelkerkes *R*^2^ = 0,302 [22]. Der Faktor „*Vermittlung eines realistischen Bildes der Inhalte vor Weiterbildungsbeginn*“ wurde nicht in die Regressionsanalyse aufgenommen, da 13 Fragebogen in diesem Punkt unvollständig waren („Missing“-Quote < 5 % angestrebt [23]).

Die Faktoren „*Hospitation <* *2* *Tage*“ (*p* = 0,002), „*geplante Op.-Einteilung*“ (*p* = 0,028) und „*Partnerschaft*“ (*p* = 0,029) sind statistisch signifikant (Tab. 1).

**Table 1:** 

Variablen	Regressionskoeffizient B	SE	Wald	*p*	Odds ratio	95 %-KI für Odds ratio Unterer Wert	95 %-KI für Odds ratio Oberer Wert
Alter < 30 Jahre	−0,654	0,369	3,15	0,076	0,52	0,252	1,071
Weibliches Geschlecht	−0,296	0,346	0,734	0,391	0,744	0,378	1,464
Radius < 150 km	−0,236	0,337	0,49	0,484	0,79	0,408	1,529
Partnerschaft	1,426	0,652	4,783	0,029	4,161	1,159	14,931
Hospitation < 2 Tage	1,487	0,472	9,917	0,002	4,422	1,753	11,155
Universitätsklinik	−0,631	0,364	3	0,083	0,532	0,261	1,087
Auswahl der Klinik durch persönliche Kenntnis	0,159	0,444	0,128	0,72	1,172	0,491	2,798
Längerfristig planbares Ausbildungskonzept	0,844	0,439	3,693	0,055	2,325	0,983	5,499
1 × im Jahr Zielvereinbarungsgespräche	−0,133	0,373	0,127	0,722	0,876	0,422	1,818
Wöchentliche, interne Fortbildung	−0,446	0,372	1,439	0,23	0,64	0,309	1,326
Förderung externer Fortbildung	−0,981	0,511	3,677	0,055	0,375	0,138	1,022
Geplante Op.-Einteilung	−0,98	0,446	4,835	0,028	0,375	0,157	0,899
Konstante	1,001	0,666	2,258	0,133	2,722	–	–

Der Faktor „*Hospitation <* *2* *Tage*“ bedingt das höchste Risiko eines Wechsels der Weiterbildungseinrichtung („odds ratio“ 4,422, 95 %-KI [1,753–11,155]), gefolgt vom Faktor „*Partnerschaft*“ (Odds ratio 4,161, 95 %-KI [1,159–14,931]).

Eine prospektive, dem Ausbildungsstand angepasste Einteilung zu Operationen minimiert die Wechselmotivation (Odds ratio 0,375).

Ohne das statistische Signifikanzniveau zu erreichen, zeigen sich weitere Faktoren, die das Risiko des Wechselns erhöhen oder vermindern. So scheinen Faktoren, wie der Beginn der Weiterbildung in einer Universitätsklinik, die persönliche Kenntnis der Klinik (z. B. durch Praktika oder die eigene Promotion), wöchentlich stattfindende interne Fortbildungsveranstaltungen sowie die zeitliche und/oder finanzielle Förderung von externen Fortbildungsprogrammen das Risiko des Klinikwechsels zu reduzieren.

Der Faktor „realistisches Bild der Inhalte vor Weiterbildungsbeginn“ wurde einer univariaten Analyse (Chi-Quadrat-Test) unterzogen. Hier zeigt sich ein signifikanter Zusammenhang zwischen „Wechsel“ und dem Faktor „realistisches Bild der Arbeitsumstände“, χ^2^ (1) = 14,92, *p* = < 0,001, φ = < 0,001 [24].

Korrespondierend hierzu ist das relative Risiko, die Klinik zu wechseln, für WA, denen vor Beginn ihrer Weiterbildung **kein** realistisches Bild der Arbeitsumstände vermittelt wurde, zweifach erhöht (2,011).

### Binär logistische Regression der Risikofaktoren für die vorzeitige Beendigung der Weiterbildung in O&U

Die Berechnung der binär logistischen Regression für das Ausscheiden aus der Weiterbildung war aufgrund der geringen Gruppengröße (*n* = 11) statistisch nicht signifikant, (χ^2^ (12) = 10,175, *p* = 0,601), mit einer nichtakzeptablen Varianzaufklärung nach Nagelkerkes *R*^2^ = 0,140 [22]. Deshalb wurde das relative Risiko mit den einzelnen Faktoren, die die Entscheidung aus der Weiterbildung O&U auszuscheiden beeinflussen, mit einer univariaten Analyse berechnet. Lediglich der Faktor „*längerfristig planbares Ausbildungskonzept*“ zeigt ein Konfidenzintervall < 1. Dieser Faktor bezieht sich auf den **Verbleib in der Weiterbildung** (RR: 1,069, 95 %-KI: 1,028–1,112) und minimiert das Abbruchrisiko.

## Diskussion

Diese Studie beschreibt erstmalig die Häufigkeit von Klinikwechseln innerhalb der Facharztweiterbildung im Fachgebiet O&U. Die Wechselquote korrelierte mit den aus der Neurochirurgie bekannten Daten (NC: 37 %, O&U: 37 %) [25]. Lediglich 8 % der Wechsel waren formal zur erfolgreichen Beendigung der Weiterbildung notwendig, da die Weiterbildung an einer Einrichtung mit eingeschränkter Weiterbildungsberechtigung begonnen wurde. Die identifizierten Einflussfaktoren für einen Klinikwechsel wie Hospitation < 2 Tage vor Beginn der Weiterbildung (RR: 2,4, 95 %-KI: 1,52–3,78) und eine prospektive OP-Einteilung (RR: 0,48, 95 %-KI: 0,28–0,82) implizieren, dass ein erheblicher Anteil der Wechsel als Reaktion auf die herrschenden Arbeits- und Weiterbildungsbedingungen erfolgt, womit sie potenziell vermeidbar wären.

Der Altersmedian der Umfrageteilnehmer*innen von 28 Jahren (Geburtsjahrgang 1992, Generation Y), genauso wie die paritätische Verteilung von weiblichen und männlichen Umfrageteilnehmer*innen, zeigt, dass die adressierten Fragestellungen für diese Generation von großer Bedeutung sind. Da ein Großteil der Weiterzubildenden gegenwärtig der Generation Y angehört, können die erhobenen Daten Weiterbildenden als Grundlage der strategischen Personalentwicklung und Ausbildungsplanung dienen.

Belastbare Rahmenbedingungen und die planbare, strukturierte Vermittlung von Weiterbildungsinhalten sind die wesentlichen Merkmale, anhand derer die Angehörigen der Generation Y eine Bewertung ihrer Weiterbildungsinstitutionen durchführen [1, 22]. Entsprechend zeigt die Studie, dass eine verbindliche OP-Planung, die den individuellen Weiterbildungsstand berücksichtigt, das Risiko für ungeplante Klinikwechsel während der Weiterbildungszeit signifikant reduzieren kann. Entscheidend ist die rechtzeitige Kommunikation der Einteilung durch den verantwortlichen OA/FA, da nur selten Motivationsdefizite oder Frustration der WA hemmend wirken [27]. Bereits 2012 zeigten Websky et al., dass die Berufszufriedenheit in der chirurgischen Weiterbildung v. a. von weiterbildungsspezifischen Aspekten wie der fähigkeitsabhängigen Einteilung zu chirurgischen Eingriffen (Odds ratio 4,2) oder der Verfügbarkeit von Trainingskursen (Odds ratio 2,7) abhängt [17]. Die zeitliche und finanzielle Förderung von Fortbildungsmaßnahmen (z. B. Wet-Lab-Training; RR: 0,67, 95 %-KI: 0,46–0,99) wird auch in der vorliegenden Studie als relevant erachtet, dabei wird eine verstärkte Supervision mit formalisiertem Feedback bei der Durchführung von Tätigkeiten von den WA gewünscht. Proske et al. demonstrieren, wie ein institutionalisiertes Mentoring mit Feedbackschleife das Potenzial hat, die Vorbereitungen für Operationen relevant zu verbessern [28]. Eine weitere Strategie, die identifizierten Forderungen in eine kompetenzbasierte ärztliche Weiterbildung, wie sie die Musterweiterbildungsordnung seit 2018 vorsieht, zu integrieren, können Entrustable Professional Activities (EPA) sein [29]. Diese beinhalten vordefinierte Kompetenzen, die vom WA zu demonstrieren sind. Erreicht ein*eine WA ein bestimmtes Niveau (Level 4) in einer EPA, befähigt dies zur selbstständigen Durchführung. Dabei steht die Kompetenz im Fokus, nicht die Anzahl an durchgeführten Tätigkeiten [30].

Wenn die zuvor genannten Bewertungsschwerpunkte der Weiterbildungsqualität über die Generationen konstant sind, ergibt sich die Frage, inwieweit die Generation Y einen anderen Beurteilungsrahmen zugrunde legt. Ein wesentlicher Hinweis ist die Tatsache, dass der Faktor „*lebt in einer Partnerschaft*“ ein signifikant erhöhtes Risiko für einen Stellenwechsel beschreibt (RR: 2,82, 95 %-KI: 1,13–7,01).

Hinsichtlich der Lebensmodelle der Generation Y werden v. a. im akademischen Umfeld vermehrt genderunabhängige „relationale“ Karriereentscheidungen im Sinne von Doppelkarrieremodellen angestrebt, d. h., es wird die Umsetzung der Vollzeitkarrieren beider Partner*innen parallel zum Familienleben versucht. Üblicherweise passiert dies aus einer relativen ökonomischen Sicherheit heraus [31].

Gleichzeitig zeigt die Generation Y eine reduzierte Loyalität gegenüber etablierten Instanzen wie dem*der Arbeitgeber*in im Allgemeinen oder der Leitung der Abteilung im Speziellen. Damit geht die **Deutungshoheit** dieser Instanzen über die Bewertung von Arbeits- und Weiterbildungsbedingungen verloren. Stattdessen wird die Bewertung durch das partnerschaftliche Umfeld zunehmend zum ausschlaggebenden Faktor. Für den*die Arbeitgeber*in bzw. den Weiterbildenden bedeutet dies, dass Initiativen zur Verbesserung der Weiterbildung nicht nur den WA, sondern mindestens in gleichem Umfang auch das partnerschaftliche Umfeld des*der WA erreichen müssen.

In diesem Zusammenhang ist die Einhaltung der vertraglich vereinbarten Arbeitszeiten ein objektiv messbares Kriterium, deren regelhafte Überschreitung im privaten Umfeld unmittelbar registriert wird. Die transparente Kommunikation von Maßnahmen zur Verbesserung der Weiterbildung und Änderungen der strukturellen Rahmenbedingungen wird zu einem wesentlichen Baustein, um Fehlwahrnehmungen zu vermeiden.

Ungeachtet dessen stellen die von der Generation Y gewünschte Planbarkeit und verbindliche Einhaltung von Arbeitszeiten unverändert eine Herausforderung dar. Die angegebene Arbeitszeit überschreitet bei 77 % der Befragten die vorgegebenen 48 Wochenstunden (EU Richtlinien von 2003) [4]. Dabei ist kein bedeutsamer Unterschied zu den von Merschin et al. [3] erhobenen Daten von 2014 erkennbar, die zeigten, dass über 80 % der Ärzt*innen in O&U mehr als 48 h pro Woche arbeiteten. Das Ziel der Arbeitszeitbegrenzung ist somit bisher nicht erreicht worden.

Auf der vorliegenden Datenbasis kann nicht geklärt werden, ob die Überschreitung der Arbeitszeiten aufgrund unzulänglicher Arbeitsorganisation zustande kommt, oder ob der zugrunde gelegte Personalbedarf den tatsächlichen Arbeitsanfall nicht abbildet. Im letztgenannten Fall können Weiterbildende durch die Definition von Personaluntergrenzen unterstützt werden, Strukturen zu etablieren, die die Einhaltung der vereinbarten Arbeitszeiten gewährleisten [32, 33].

Das Geschlecht hat in der vorliegenden Studie keinen signifikanten Einfluss auf das Wechseln oder Ausscheiden aus der Weiterbildung und unterscheidet sich somit von den erhobenen Daten in der NC [25]. Die Parität der Geschlechterverteilung der Teilnehmer*innen, bei gleichzeitig höherem Männeranteil in der Gesamtpopulation der WA, lässt vermuten, dass die abgefragten Thematiken für Chirurginnen allerdings eine höhere Relevanz haben als für die männlichen Kollegen. Dabei haben relevante Themen wie die verlässliche Einhaltung der Arbeitszeiten und das rechtzeitige Erscheinen des Dienstplanes Schnittmengen mit den geschlechtsunabhängigen Forderungen der Generation Y. Die in dieser Generation gelebten Partnerschaftsmodelle zeigen, dass Themen, die in der Vergangenheit hauptsächlich mit der Lebenswirklichkeit von Chirurginnen assoziiert wurden, mittlerweile tatsächlich genderunabhängige Themen einer Generation sind.

Gleichzeitig darf nicht ignoriert werden, dass im 8. Semester 39 % der Medizinstudentinnen O&U als Wunschfach angeben, wohingegen nach dem Praktischen Jahr nur noch 13 % diesen Wunsch äußern. Begründet wird der Interessenverlust mit den Arbeitszeiten, der Unvereinbarkeit mit der Familienplanung sowie dem Umstand, dass O&U ein männerdominierter Fachbereich sei [7, 20]. Chirurginnen gewichteten in einer Umfrage im Jahr 2010 die Faktoren Work-Life-Balance und Familienleben höher als männliche Kollegen [34].

Alle skizzierten Maßnahmen werden Wechsel in der Weiterbildung nicht vollständig verhindern können. Vielmehr ist abschließend zu diskutieren, welches Potenzial gesteuerte, mandatorische Wechsel für die Gestaltung der Weiterbildung, wie sie z. B. in der schweizerischen Musterweiterbildungsordnung bereits vorgesehen sind, haben [35]. Wechsel zwischen Krankenhäusern unterschiedlicher Versorgungsstufen können unterschiedliche Dienstmodelle ermöglichen, den Zugang zu Fortbildungskonzepten steigern und das operative Ausbildungsspektrum erweitern. Das Ziel muss eine gesteigerte Zufriedenheit mit den Weiterbildungsbedingungen sein.

## Limitationen

- Es liegen keine Daten zu den tatsächlichen Ausbildungsleistungen (z. B. OP-Katalog) der befragten WA vor.
- Die Umfrage erreichte potenziell nur 39 % der bundesweit geschätzt im Fach O&U tätigen Weiterbildungsassistent*innen. Eine Rücklaufquote von 11 %, was 4 % der geschätzten Grundgesamtheit entspricht, limitiert die Generalisierbarkeit der Aussagen der Studie [14]. Es können jedoch entsprechende Trends beschrieben werden.
- Selection bias durch die Schwierigkeit, vollständig aus der Weiterbildung ausgeschiedener WA zu erreichen.

## Ausblick

In der Vergangenheit war das frühestmögliche Ablegen der Facharztprüfung das wesentliche Qualitätskriterium einer gelungenen Weiterbildung. Vor dem Hintergrund der hier dargestellten Entwicklung wird klar, dass gegenwärtig sowohl die Bewertungsmaßstäbe als auch die bewertenden Instanzen (partnerschaftliches Umfeld) einem grundlegenden Wandel unterworfen sind.

In der aktuellen Weiterbildungsgeneration werden aus empfundener Unzufriedenheit mit den Weiterbildungsbedingungen Konsequenzen in Form von Arbeitsplatzwechseln gezogen. Das stellt eine Herausforderung für die Arbeitgeber*innen dar.

Die Entwicklung von Arbeitszeitmodellen, die dem*der WA die Möglichkeit eröffnen, die persönliche Arbeitsbelastung ausbildungs- und lebensphasengerecht zu steuern, bildet die Grundlage für eine Bindung der Generation Y an das Fachgebiet, die Weiterbildung und den*die Arbeitgeber*in (**Empowerment**). Zukünftige Tarifverträge müssen über ihre Schutzfunktion hinaus die Diversität der Lebens- und Arbeitsmodelle in O&U abbilden. Ein Flächentarifvertrag, der weder die individuellen Bedürfnisse der WA noch die Notwendigkeiten der weiterbildenden Abteilungsleiter und deren Klinikträger abbildet, kann die Generation Y in der Umsetzung ihrer Lebensmodelle behindern.

Die letztgenannten Überlegungen setzen voraus, dass die WA sich tatsächlich für ein belastbares Lebens- und Karrieremodell entscheiden. In Zukunft wird die Personalentwicklung einer Klinik den Lebensmodellen der WA und Mitarbeiter*innen folgen müssen; um im Gegenzug von den WA und Mitarbeiter*innen, die zur Aufrechterhaltung einer qualitativ nachhaltigen Patientenversorgung zwingend erforderliche Loyalität zu erhalten.

In der praktischen Umsetzung von Aus- und Weiterbildung haben diese Entwicklungen Konsequenzen, die zum jetzigen Zeitpunkt kaum absehbar sind. Insbesondere die Vermittlung und der Erhalt von Skills werden das tradierte Lehr- und Lernmodell des „Schau hin und sei dabei“ verlassen müssen. Die punktuell verfügbaren und didaktisch ausgereiften Wet-Lab-Weiterbildungsformate sind ein Schritt in die richtige Richtung, die durch Konzepte wie z. B. spezifische Mentoring-Programme, die schwangerschaftsbedingten Verzögerungen der Ausbildung, genauso wie Skill-Verluste, gezielt adressieren und auffangen [36].

## Fazit für die Praxis

Die Weiterbildung in O&U stellt hohe fachliche und psychische Anforderungen an die WA, die ein hohes Maß an Resilienz erfordern. Neben den oben genannten grundsätzlichen Veränderungen gilt schon jetzt für die tägliche Praxis:

Enttäuschung vernichtet Resilienz. Enttäuschungen können vermieden und damit das Risiko eines Klinikwechsels reduziert werden durch:

- Hospitationen von mehr als einem Tag Dauer vor Weiterbildungsbeginn,
- Weiterbildungsbedingungen, die vereinbar sind mit den Anforderungen von modernen Partnerschaften (e. g. Einhaltung der Arbeitszeiten, Flexibilisierung/Teilzeitmodelle).

Verlässlichkeit und wahrgenommener Lernfortschritt schaffen Resilienz durch:

- Verbindlichkeit strukturierter Weiterbildung (z. B. OP-Einteilung gemäß dem Weiterbildungsstand oder Nutzung von EPA zur einheitlichen Definition des Erwartungshorizontes),
- Implementierung extracurriculärer und intramuraler Fortbildungsformate (z. B. Wet-Lab).

Zusammenfassend scheinen die Bedürfnisse der WA zunehmend homogener zu werden und entwickeln sich weg von einer reinen Genderthematik hin zu einem Generationsthema.

### Supplementary Information





## Abkürzungen

KI: Konfidenzintervall
MW: Mittelwert
NC: Neurochirurgie
O&U: Orthopädie & Unfallchirurgie
Survey: Onlinefragebogen
WA: Weiterbildungsassistent*innen
