# Supplementary material for: Analysis of risk factors for dropping out of residency in orthopedics and trauma surgery
Source: Unfallchirurgie (Heidelb). 2022 Nov 10;126(10):788–98. [Article in German] doi: 10.1007/s00113-022-01249-x (PMC10520118; doi:10.1007/s00113-022-01249-x)
Supplement: Supplementary file 1 [file 113_2022_1249_MOESM1_ESM.pdf]

OUT VORAUS Survey Biografie

1. Wie alt waren Sie beim Beginn Ihrer orthopädischen/unfallchirurgischen Facharztweiterbildung? (**Alter in Jahren, z.B. 27**)
   - *Skala: 20-67*

2. Im welchem **Ausbildungsjahr** der Facharztweiterbildung befinden Sie sich aktuell?

   a) *1.-2. Jahr (Common Trunk)*
   b) *3.-4. Jahr (Special Trunk)*
   c) *5.-6. Jahr (Special Trunk)*
   d) *Bereits Facharzt*ärztin*

3. Welchem **Geschlecht** fühlen Sie sich zugehörig?

   a) *Weiblich*
   b) *Männlich*
   c) *Divers*

OUT VORAUS Survey
Privatleben

4. Sind Sie in der Region (<150km Pendlerdistanz) aufgewachsen, in der Sie arbeiten bzw. gearbeitet haben?

- *Ja/Nein*

5. Fühlen Sie sich in der Stadt wohl, in der Sie arbeiten bzw. gearbeitet haben?

- *Ja/Nein*

6. Haben Sie **Freizeitaktivitäten**, die sie regelmäßig wahrnehmen (mindst. >1x pro Woche, 1h Dauer)? (Mehrfachnennung möglich)

a) Keine
b) Sportliche Aktivität
c) Spielen von Musikinstrumenten
d) Soziales Engagement
e) Sonstiges (bitte angeben)

7. Haben Sie oder nehmen Sie in Ihrer Freizeitaktivität (Frage 6) an vergleichenden **Wettbewerben** teilgenommen?

- *Ja/Nein*

8. Befanden Sie sich zu irgendeinem Zeitpunkt der Weiterbildung in einer **festen Partnerschaft**?

- *Ja/Nein*

9. **Falls** ja, wo lebt/lebte Ihr Partner?

a) Zusammen im selben Haushalt
b) Weniger als 50km entfernt
c) Mehr als 50km entfernt

OUT VORAUS Survey Initiale Karriereplanung

10. Mit welcher **beruflichen Zielsetzung** haben Sie die Facharztweiterbildung begonnen? (Mehrfachnennung möglich)

   a) *Niederlassung als Facharzt (ambulanter Sektor)*
   b) *Karriere im Krankenhaus (stationäre Versorgung)*
   c) *Wissenschaftliche Karriere*
   d) *Berufliche Tätigkeiten außerhalb der direkten Patientenversorgung (z.B. Krankenhausmanagement, Versicherung, MDK, etc.)*
   e) *Zu Beginn keine definierte Zielsetzung*

11. **Wenn wissenschaftliche Karriere**: Wann führen Sie die wissenschaftliche Tätigkeit durch?

   a) *Überwiegend in meiner Freizeit*
   b) *Überwiegend in der Arbeitszeit (temporäre Freistellung)*
   c) *Während einer Forschungsrotation*

12. Wo haben Sie zu Beginn der Weiterbildung in O&U Ihren **größten Interessensschwerpunkt** gesehen?

   a) *Notfallversorgung (Präklinisch/ Schockraumversorgung*
   b) *Allgemeine Traumatologie*
   c) *Alterstraumatologie*
   d) *Allgemeine Orthopädie*
   e) *Kinderorthopädie*
   f) *Sporttraumatologie/-orthopädie*
   g) *Keinen Schwerpunkt*

13. Erfolgte vor der Einstellung eine **Hospitation**?

a) ***1 Tag*** *Dauer*
b) *Bis zu* ***1 Woche*** *Dauer*
c) *Bis zu* ***1 Monat*** *Dauer*
d) ***PJ/Famulatur*** *in der Klinik*
e) *Nein*

14. Mit welchen Berufsgruppen des Krankenhauses haben Sie vor Ihrer Einstellung ein Gespräch geführt? (Mehrfachnennung möglich)

a) *Mit anderen* ***Assistenzärzt*innen***
b) *Mit dem* ***Oberarzt*ärztin***
c) *Mit dem/der* ***Chef*in***
d) *Mit der* ***Vertretung der Personalabteilung***
e) *Kein formales Gespräch*

15. In welcher klinischen Versorgungsstufe haben Sie die Weiterbildung begonnen?

a) *Universitätsklinikum*
b) *Kommunales Krankenhaus*
c) *Krankenhaus mit kirchlichem Träger*
d) *Krankenhaus mit privatem Träger*
e) *Klinik mit eingeschränkter Weiterbildungsermächtigung*
f) *Spezialklinik (z.B. Rehabilitationskliniken)*
g) *Niederlassung/ Praxis (ambulanter Bereich)*

16. Warum haben Sie die Einrichtung für Ihre Weiterbildung ausgewählt? (**wichtigster Grund**)

a) *Kenntnis der Klinik durch* ***persönliche Erfahrungen*** *vor Ort (Studium, Famulatur, Praktisches Jahr)*
b) *Aufgrund einer* ***persönlichen Empfehlung***
c) *Aufgrund der* ***geografischen Lage*** *des Weiterbildungsortes*
d) *Kenntnis der Klinik* ***durch Ihre Doktorarbeit***
e) *Aufgrund des* ***fachlichen Klinikprofils***
f) *Offenes* ***Stellenangebot***
g) *Sonstiges (bitte angeben)*

OUT VORAUS Survey
Reale Arbeitsbedingungen

17. **Wie viele** Weiterbildungsassistent*innen arbeiten in ihrer Klinik

- *Skala: 0-80 (Anzahl der Weiterbildungsassistent*innen)*

18. Gab/gibt es in Ihrer Klinik eine **Vertretung der Assistent*innen**?

- *Ja/Nein*

19. Wie hoch ist die derzeitige **effektive wöchentliche Arbeitszeit**?

a) <42 Stunden
b) 42 – 48 Stunden
c) 49 – 54 Stunden
d) >54 Stunden

20. Wie viele **Bereitschaftsdienste** führen Sie aktuell **pro Monat** durch? (inkl. Ruf- und Spätdiensten)

a) Keine
b) 1-2
c) 3-4
d) 5-6
e) 7-8
f) >8

21. Wie viel Prozent (%) der wöchentlichen Arbeitszeit sind sie **im Operationssaal**?

a) <20%
b) 21-40%
c) 41-60%
d) >60%

22. Wie viel Prozent (%) der wöchentlichen Arbeitszeit verwenden Sie durchschnittlich für die „**Patientenversorgung**“ (Station, Ambulanz, Sprechstunde)?

a) <20%
b) 21-40%
c) 41-60%
d) >60%

23. Wie viel Prozent (%) der wöchentlichen Arbeitszeit verwenden Sie durchschnittlich für „**Bürokratie**“/ "**nicht-ärztliche" Tätigkeiten**?

a) <20%

b) *21-40%*
c) *41-60%*
d) *>60%*

24. Gibt es in Ihrer Klinik ein **verbindliches Ausbildungscurriculum** (z.B. Rotationsplan)?

a) *Ja, als langfristiges, planbares Konzept*
b) *Ja, aber regelhaft kurzfristige Änderung (eingeschränkte Planbarkeit)*
c) *Kein strukturiertes Ausbildungskonzept vorhanden*

25. Wie oft finden **Zielvereinbarungsgespräche** in Ihrer Klinik statt?

a) *Halbjährig*
b) *1x im Jahr*
c) *Unregelmäßig (< 1x im Jahr)*
d) *Gar nicht*

26. Gibt es in ihrer Klinik regelmäßige **Fortbildungsveranstaltungen** für die Assistent*innen (z.B. Röntgenbesprechung/ wöchentliche Fortbildungen/ Medizinproduktschulungen/ M&M Konferenzen etc.)?

a) *Regelmäßig (mindestens 1x/ Woche)*
b) *Unregelmäßig (weniger als 1x/ Woche)*
c) *Keine*

27. Wird die Teilnahme an **klinik-externen Kursprogrammen** finanziell und/oder zeitlich gefördert (**ATLS; AE- und AO-Kurse**)?

- *Ja/Nein*

28. Finden Anteile Ihrer Ausbildung **simulatorgestützt** oder im **Wet-Lab** statt?

- *Ja/Nein*

29. Welcher Faktor beeinflusst in Ihrer Klinik primär die **Einteilung als Operateur*in?**

a) *Persönliches Sympathieverhältnis zum OP-Plan erstellenden OÄ/OA/Chef*in*
b) *Ausbildungsstand*
c) *Entsprechend des Weiterbildungsplanes*
d) *Tagesaktuelle personelle Verfügbarkeit*
e) *Wissenschaftliche Tätigkeit*
f) *Willkürlich*

30. Werden klinikinterne, gesellschaftliche Veranstaltungen durchgeführt? (Mehrfachnennung möglich)

a) *Nein, nie*

b) *Weihnachtsfeier*
c) *Gemeinsame Ausflüge*
d) *Assistent*innentreffen*

OUT VORAUS Survey

Abschließende Fragen

31. Sie haben während Ihre Facharztweiterbildung in O&U...

*a) Die Klinik **nicht** gewechselt.*
*b) Aus **fachlichen Gründen** die Klinik gewechselt*
*c) Aus **persönlichen Gründen** die Klinik gewechselt.*
*d) Die **Richtung** der Facharztweiterbildung **geändert***
*e) Die Weiterbildung **dauerhaft beendet***

32. Kennen Sie **Kolleg*innen** aus Ihrer Klinik, die die **Facharztweiterbildung abgebrochen** haben?

a) Nein
b) Ja, wieviele? (Zahl)

33. Kennen Sie **Kolleg*innen** aus Ihrer Klinik, die die **Klinik aus fachlichen Gründen gewechselt** haben?

a) Nein
b) Ja, wie viele (Zahl)

OUT VORAUS Survey
Persönliche Einstellung

Wir bitten Sie, jede der nun folgenden Aussagen einzeln auf Ihre Relevanz für den erfolgreichen Abschluss der Facharztweiterbildung in O&U zu bewerten.
(**1 gar keine Zustimmung – 6 maximale Zustimmung zur gemachten Aussage**) *(In Form von Likert-Skalen)*

34. Die Einhaltung von tariflich geregelten Arbeitszeiten in der Weiterbildung ist mir wichtig! (**Life-Work-Balance**)

35. Das **rechtzeitige Erscheinen** eines gültigen **Dienstplanes** ist mir wichtig! (mindestens 4 Wochen vorher)

36. Mir ist es wichtig, dass die Klinikleitung regelmäßig eine **klare Erwartungshaltung** an mich formuliert!

37. Eine **breite**, den unmittelbaren fachlichen Schwerpunkt von O&U übertreffende **chirurgische Ausbildung** ist mir wichtig! (z.B. allgemein-, viszeral- oder gefäßchirurgische Ausbildungsabschnitte)

38. Auch die Facharztweiterbildung sollte bereits eine **fachliche Schwerpunktbildung** nach persönlichen Wünschen ermöglichen!

39. Die direkte, persönliche Anleitung bei Tätigkeiten ist mir wichtig! **(Supervision)**

40. Das Vorhandensein von einheitlichen, klinik-internen Standards **(SOP´s)** zur Patientenversorgung ist mir wichtig!

41. Regelmäßige, **klinik-interne Fortbildungen** sind mir wichtig! (z.B. Röntgenbesprechung/ wöchentliche Fortbildungen/ Medizinproduktschulungen/ M&M Konferenzen etc.)

42. Die Unterstützung bei dem Besuch von **klinik-externen Ausbildungscurricula** (z.B. ATLS, AO- und AE-Kursen) ist mir wichtig! (**zeitlich und/ oder finanziell**)

43. Die moderne orthopädisch/ unfallchirurgische Weiterbildung soll **praktische Ausbildungsanteile an realitätsnahen Modellen** (Wet-Lab) beinhalten!

44. Wurde Ihnen vor dem Antritt der Weiterbildung ein realistisches Bild der Arbeitsumstände vermittelt?

- *Ja/Nein*

45. Werden Sie oder haben Sie zum Ende Ihrer regulären Facharztweiterbildungszeit die Ziele des Facharztkataloges real erreicht?

- *Ja/Nein*

46. Würden Sie abschließend die Facharztweiterbildung „**Orthopädie und Unfallchirurgie**" als Berufsziel **weiterempfehlen**?

a) *Uneingeschränkt*
b) *Ja, in meiner aktuellen Klinik*
c) *Ja, in einer anderen Klinik*
d) *Überhaupt nicht*
e) *Weiß nicht*

OUT VORAUS Survey

Informationen zur Beendigung der Weiterbildung

47. Wann wurde die Weiterbildung abgebrochen?

a) *In der **Probezeit***
b) *In den ersten **6 Monaten** der Facharztausbildung*
c) *Im **1. Jahr** der Facharztausbildung*
d) *Im **2. Jahr** der Facharztausbildung*
e) *Im **3. Jahr** der Facharztausbildung*
f) *Im **4. Jahr** der Facharztausbildung*
g) *Im **5. Jahr** der Facharztausbildung*
h) *Im **6. Jahr** der Facharztausbildung*

48. Wie erfolgte die Kündigung?

a) *Spontan (**in der Probenzeit**), ohne konkrete weitere berufliche Perspektive*
b) *Spontan (**fristlos**), ohne konkrete weitere berufliche Perspektive*
c) *Geplant (**fristgerecht**), mit konkreter weiterer beruflicher Perspektive*

49. In welchem Bereich lagen die Gründe der Kündigung?

a) *Persönliches Umfeld*
b) *Arbeitsumfeld*

50. Aus welchem Grund/Gründen haben Sie die Weiterbildung abgebrochen bzw. welche Kritikpunkte sehen Sie in Ihrer derzeitigen Weiterbildung? (Mehrfachnennung möglich)

a) *Starke Arbeitsbelastung während der regulären Arbeitszeit*
b) *Überstunden, Mangel an Freizeit*
c) *Psychischer Stress durch fatale Krankheitsbilder und Verläufe*
d) *Mangelnde operative Ausbildung*
e) *Psychischer Stress durch hohe Verantwortung*
f) *Zu viel administrative Tätigkeit*
g) *Zu geringes Gehalt*
h) *Spannungen im Team*
i) *Hierarchie in der Klinik/Umgangston*
j) *Belastung durch Forschungsprojekte*
k) *Fehlende strukturierte Weiterbildung*
l) *Benachteiligung auf Grund ihres Geschlechts*
m) *Benachteiligung auf Grund ihrer Herkunft*
n) *Benachteiligung auf Grund von Familiengründung/-planung bzw. mangelnde Familienkompatibilität der Weiterbildung*

51. Aus welchem Grund/Gründen haben Sie die Weiterbildung abgebrochen bzw. welche Kritikpunkte sehen Sie in Ihrer derzeitigen Weiterbildung? (Mehrfachnennung möglich)

a) Starke Arbeitsbelastung während der regulären Arbeitszeit
b) Überstunden, Mangel an Freizeit
c) Psychischer Stress durch fatale Krankheitsbilder und Verläufe
d) Mangelnde operative Ausbildung
e) Psychischer Stress durch hohe Verantwortung
f) Zu viel administrative Tätigkeit
g) Zu geringes Gehalt
h) Spannungen im Team
i) Hierarchie in der Klinik/Umgangston
j) Belastung durch Forschungsprojekte
k) Fehlende strukturierte Weiterbildung
l) Benachteiligung auf Grund ihres Geschlechts
m) Benachteiligung auf Grund ihrer Herkunft
n) Benachteiligung auf Grund von Familiengründung/-planung bzw. mangelnde Familienkompatibilität der Weiterbildung

Ende der Umfrage

Vielen Dank für Ihre Teilnahme!

58. Zur Teilnahme an der Verlosung der Buchgutscheine können sie nun noch freiwillig Ihre Kontaktdaten eingeben:

Name

E-Mail-Adresse
